# Total and Metabolically Active Microbial Community of Aerobic Granular Sludge Systems Operated in Sequential Batch Reactors: Effect of Pharmaceutical Compounds

**DOI:** 10.3390/toxics9050093

**Published:** 2021-04-23

**Authors:** Barbara Muñoz-Palazon, Aurora Rosa-Masegosa, Miguel Hurtado-Martinez, Alejandro Rodriguez-Sanchez, Alexander Link, Ramiro Vilchez-Vargas, Alejandro Gonzalez-Martinez, Jesus Gonzalez Lopez

**Affiliations:** 1 Faculty of Pharmacy, University of Granada, Campus de Cartuja, s/n, 18071 Granada, Spain; aurorarm@ugr.es (A.R.-M.); miguelhm@ugr.es (M.H.-M.); jgl@ugr.es (J.G.L.); 2 Institute of Water Research, University of Granada, C/Ramón y Cajal, 4, 18071 Granada, Spain; 3 Department of Horticulture and Landscape Architecture, Purdue University, 625 Agriculture Mall Drive, West Lafayette, IN 47907, USA; rodri719@purdue.edu; 4 Department of Gastroenterology, Hepatology, and Infectious Diseases, Otto von Guericke University Hospital Magdeburg, 39120 Magdeburg, Germany; alexander.Link@med.ovgu.de (A.L.); ramiro.vilchez@med.ovgu.de (R.V.-V.)

**Keywords:** aerobic granular sludge, pharmaceutical compounds, wastewater, total microbial community, active microbial community, qPCR

## Abstract

Two aerobic granular sludge (AGS) sequential batch reactors were operated at a mild (15 °C) temperature for 180 days. One of those bioreactors was exposed to a mixture of diclofenac, naproxen, trimethoprim, and carbamazepine. The AGS system, operating under pressure from emerging contaminants, showed a decrease in COD, BOD_5_, and TN removal capacity, mainly observed during the first 100 days, in comparison with the removal ratios detected in the control bioreactor. After an acclimatisation period, the removal reached high-quality effluent for COD and TN, close to 95% and 90%, respectively. In the steady-state period, trimethoprim and diclofenac were successfully removed with values around 50%, while carbamazepine and naproxen were more recalcitrant. The dominant bacterial OTUs were affected by the presence of a mixture of pharmaceutical compounds, under which the dominant phylotypes changed to OTUs classified among the *Pseudomonas, Gemmobacter*, and *Comamonadaceae*. The RT-qPCR and qPCR results showed the deep effects of pharmaceutical compounds on the number of copies of target genes. Statistical analyses allowed for linking the total and active microbial communities with the physico-chemical performance, describing the effects of pharmaceutical compounds in pollution degradation, as well as the successful adaptation of the system to treat wastewater in the presence of toxic compounds.

## 1. Introduction

The worldwide concern about pharmaceutical compounds (PCs), which are named “emerging contaminants”, has been growing during recent decades. The widespread detection of pharmaceuticals in terrestrial and aquatic systems has engendered significant scientific and regulatory concerns [1]. These products are widely used but bring harmful effects to the environment. Most of the PCs are not completely degraded by human or animal bodies, thus being discharged, by means of excretion, mostly unaltered or as an active metabolite going into wastewater treatment plants (WWTPs), finally ending up in water ecosystems. The PCs mainly reported in wastewaters include antibiotics, anti-inflammatories, and antiepileptics, among others. Even at low concentrations, the PCs could affect the composition of the microbial communities that comprise the secondary treatment technology, and hence disturb the metabolic networks in the population [2]. The most abundant pharmaceutical compounds analysed in wastewater from the biggest hospital of the south of Spain, specifically in Granada, were carbamazepine, trimethoprim, naproxen, and diclofenac, as described by Olicón-Hernandez et al. [3]. 

Carbamazepine (CBZ) is a highly recalcitrant habitual anticonvulsant drug. Currently, it is used as an indicator of anthropogenic impacts on water bodies [4]. Trimethoprim (TMP) is an antibiotic belonging to chemotherapeutic agents acting on dihydrofolate reductase, inhibiting the synthesis of tetrahydrofolic acid, which is the most often-used veterinary medicine [5]. TMP has been habitually detected in wastewater [6], demonstrating that conventional wastewater treatment processes do not effectively remove it. The presence of certain antibiotic concentrations in the natural environment, especially in water bodies, causes adverse and damaging effects, including bacteria developing antibiotic resistant genes [7]. Naproxen (NPX) is a commonly used drug that possesses anti-inflammatory and analgesic activities, namely a pain killer, due to its ability to inhibit cyclooxygenase enzymes that promote inflammation [8]. Diclofenac (DCF) is also a weak organic acid that belongs to the group of non-steroidal anti-inflammatory drugs, which play an anti-inflammatory and analgesic role, inhibiting the synthesis of prostaglandins [9]. The high concentrations of NPX and DCF in natural water bodies could be caused by the polar nature of both, and they easily escape the wastewater treatment process and are discharged into the environment [10].

Aerobic granular sludge (AGS) technology has been extensively studied during recent years because it is a system with several advantages in comparison with conventional activated sludge (CAS). The aerobic granular sludge is a complex matrix of microorganisms embedded in a complex of extracellular polymeric substances (EPSs) that act as a union bridge, providing particles of self-immobilised microorganisms with no supporting carrier [11]. The hydrodynamic circular motion promotes the formation of spherical sludge aggregates, which allow the coexistence of aerobic, anoxic, and anaerobic layers, where several metabolic pathways are carried out. The AGS technology has numerous advantages in comparison with other biological treatment technologies, such as nutrient removal in the same chamber under the same operational conditions, excellent settleability, resilience to shocks against toxic compounds, high biomass retention, and low, or absence of, waste biomass production [12,13]. Additionally, AGS has a high efficiency to remove, adsorb, or transform other compounds such as particulate matter, pharmaceutical compounds, nuclear waste, olive washing water, or textile wastewater, amongst others [11,14]. The treatment of these industrial wastewaters, in which there are high loads of toxic compounds, is possible due to the mass transfer limitations to protect the microorganisms in the internal layers from shock loading with different compounds of differing natures [4]. In fact, the AGS technology has acquired relevance in wastewater treatment areas for removing emerging contaminants. Finally, most studies about biological technologies linking the operational conditions, physico-chemical determinations, and the microbial communities understand that the contribution to some actions is strictly related to the relative or absolute abundance in the population studied by target genes. However, the study of metabolically active microorganisms makes a deep change in the conception of the roles that microorganisms play in wastewater treatment. Consequently, there is a lack of knowledge when the biological analysis is based exclusively on DNA analysis, while that of RNA material is ignored. There are few studies that have identified and quantified both of the nucleic acids in biological wastewater technologies, but, nevertheless, they are very valuable [15,16].

Therefore, the main objective of this study is to evaluate the effect of a mixture of pharmaceutical compounds on the physico-chemical performance and granular stability (1); and on the total and metabolically active microbial community (2); and quantify the removal capability for the selected emergent contaminant (3).

## 2. Materials and Methods

### 2.1. Design of Bioreactors, Configuration, and Operation

Two AGS reactors were implemented using cylindrical columns with a height of 45 cm and a diameter of 9 cm (Figure S1), operated as sequential batch reactors (SBRs). The operational volume was 2.5 L, of which 60% was exchanged per cycle. The hydraulic retention time was 6.6 h and the cycles consisted of: 33 min for feeding with raw water, 230 min of continuous aeration, 3 min of settling, and 4 min for effluent discard. The air was introduced by fine bubbles at the bottom using a 3 L min^−1^ flow rate. The bioreactors were kept at 15 °C during the 180 days of experimentation. The pH was monitored at a value of 7.6 ± 0.2, and the dissolved oxygen was close to saturation.

Both bioreactors were firstly inoculated with 1 L of granular biomass from an aerobic granular sludge system operated in a sequential batch reactor for treating synthetic wastewater at a lab scale [17]. The bioreactors were fed using synthetic wastewater, simulating urban sewage with the following composition: CH_3_COONa 0.9 g L^−1^, NH_4_Cl 0.25 g L^−1^, MgSO_4_·7H_2_O 0.1 g L^−1^, K_2_HPO_4_ 0.085 g L^−1^, KCl 0.04 g L^−1^, and KH_2_PO_4_ 0.03 g L^−1^ [18]. In every feeding period, wastewater was introduced from the top of the reactors by peristaltic pumps (Watson Marlow, UK). Four pharmaceuticals (diclofenac, naproxen, trimethoprim, and carbamazepine) were added to one of the two bioreactors at a concentration of 25 µM [19]. The concentration of these drugs added in the influent, expressed in mg L^−1^, were for diclofenac 7.403696 mg L^−1^, for naproxen 5.756449 mg L^−1^, for trimethoprim 7.258 mg L^−1^, and for carbamazepine 5.906725 mg L^−1^. The other AGS was used as a control without pharmaceutical compounds in order to evaluate their effect on the physico-chemical performance, granular stability, and microbial community. The control bioreactor (CB) and pharmaceutical-added bioreactor (PB) were used to compare the evolution and development of the systems and their microbial communities.

### 2.2. Physico-Chemical Determinations

Chemical oxygen demand (COD) and biological oxygen demand (BOD5) were analysed according to standard methods described by APHA [20]. The COD and BOD_5_ were expressed as removal ratio, based on the concentration in the influent and effluent. In addition, nitrogen ions, NH_4_^+^, NO_3_^−^, and NO_2_^−^ were analysed using an ion chromatograph (Metrohm Ion Chromatograph, AG, Switzerland). The efficiency in terms of nitrogen was expressed as total nitrogen removal, and the calculation was done using the difference in concentrations of the nitrogen compounds (NH_4_^+^, NO_3_^−^, and NO_2_^−^), expressed as mg-N L^−1^ of the influent and effluent. The settling velocity was measured using a measuring cylinder by recording the falling time of a single granule freely dropped from a certain height in water, following the protocol described by Laguna et al. [21], and the mean size of granules was also measured using a scalemeter [22]. Mixed liquor suspended solids (MLSSs) were measured 3 times a week according to APHA [20]. The dissolved oxygen and pH in the bioreactors were monitored by a Crison Oximeter and Crison pH meter, respectively.

### 2.3. Pharmaceutical Concentration Determination

The samples containing pharmaceutical compounds were analysed. For that, samples were previously pH adjusted with 0.1 N HCl until they reached pH 4.5. Columns were pre-conditioned with an elution of 8 mL of methanol and 8 mL of HPLC water, consecutively. Then, 100 mL of the collected samples were passed through an extraction column in a solid phase (SPE) Oasis HLB cartridge (200 mg, Milford, CT, USA). Finally, the cartridges were washed with Milli-Q water (10 mL) and air dried. The extraction was carried out with a vacuum system.

A sample volume of 10 μL was injected, eluting with a mobile positive ionisation phase composed of fractions of H_2_O–formic acid 0.1% (A) and acetonitrile (B), and a mobile negative ionisation phase composed of the fractions H_2_O–NH_3_ 0.1% (A) and methanol (B). The detection system was a Triple Quadruple XEVO-TQ-XS Waters spectrometer (Milford, MA, USA). The concentration of the candidate PCs was obtained by interpolation of standards curves. Pharmaceutical standards were dissolved in acetonitrile.

### 2.4. Biomass Collection and Nucleic Acid Extraction

With the aim to obtain representative samples of biomass, 100 mL of granules were taken during the aeration period. Subsequently, sterile saline solution 0.9% was added to the samples, which were sonicated for 10 min and centrifuged at 8000 rpm at 4 °C for 10 min. Then, supernatants were discarded, and the pellet was preserved at −80 °C. Additionally, RNA Protect reagent (QIAGEN) was added to biological samples and they were kept at −80 °C in order to maintain the quality of the RNA.

Afterwards, DNA extraction was performed on the pellets kept at −80 °C using the FastDNA SPIN Kit for Soil and FastPrep equipment (MP Biomedicals, Solon, OH, USA) following the protocol given by the manufacturer.

RNA extraction was performed using a FastRNA Blue Kit (MP-Biomedical, USA) following the manufacturer’s protocol. Then, RNA samples were digested using the TURBO DNA-free Kit (Ambion, Life Technologies Corporation, CA, USA) and the remaining digestates were purified employing the RNeasy Mini Kit (Qiagen, Hamburg, Germany). The reverse transcription of RNA to cDNA was done using Superscript III Reverse Transcriptase (Invitrogen), following the procedure described by the manufacturer. Finally, DNA and cDNA extracted libraries were constructed for next-generation sequencing analysis using specific primers to amplify Bacteria and Archaea domains.

### 2.5. Massive Parallel Sequencing Procedure

The next-generation sequencing was done using Illumina MiSeq equipment and Illumina MiSeq Reagent v3, sequencing 300 paired-end nucleotides. Pools of nucleic acids were amplified twice per sample for each pair of primers and the mix. The pair of primers Bacteria807F (5′-GGATTAGATACCCBRGTAGTC-3’) and Bacteria1050R (5’-TAGYTGDCGACRRCCRTGCA) was used for the amplification of the hypervariable region V5-V6 of 16S rRNA of bacteria [23]. The PCR conditions of the next-generation sequencing process were the following: 2 min at 95 °C; then 30 cycles of: 30 s at 95 °C, 30 s at 57 °C, and 1 min at 72 °C; then 5 min of final elongation at 72 °C. The primers used for detection of the Archaea domain were U519F (5’-CAGYMGCCRCGGKAAHACC-3’) and Arch806R (5’-GGACTACNSGGGTMTCTAAT-3’) for the amplification of V4 of 16S rRNA of archaea [24]. The PCR conditions of the next-generation sequencing process were the following: 5 min at 95 °C; then 40 cycles of: 30 s at 95 °C, 30 s at 56 °C, and 1 min at 72 °C; then 5 min of final elongation at 72 °C.

### 2.6. Bioinformatics Pipeline

Data from next-generation sequencing were processed using mothur software v1.44.3 [25]. Firstly, forward and reverse paired-end reads were merged into contigs for all raw samples using Needleman alignment conditions, and assuming an ambiguous base in the overlap region when nucleotides in the same position had a difference in the Phred score lower than 0. The contigs were then subjected to quality trimming to remove sequences with homopolymers longer than 8 bp, and any ambiguous bases [26]. The remaining contigs were aligned using Needleman conditions through the k-nearest-neighbour method with k-mer size of 8 bp against the SILVA SEED v123 database. Then, the contigs that did not align correctly at the forward and reverse positions of the used primers were removed for both domains. Chimerical contigs were de novo detected using the VSEARCH algorithm [27]. Then, the remaining contigs were taxonomically classified against the MiDAS [28] database for bacteria and the MiDAS and SILVA databases for archaea, using the k-nearest-neighbour algorithm and the k-mer search method with a k-mer size of 8 bp. The contigs that were not successfully classified at the phylum level in each of the domains were removed from the analyses. Then, the remaining contigs were clustered into OTUs using a distance greedy algorithm with a similarity cutoff of 3% for bacteria, and 5% for archaea.

### 2.7. Absolute Quantification of Total and Metabolically Active Microorganisms

The absolute abundances of total and metabolically active communities of bacteria, archaea, and fungi of the granular sludge were analysed by qPCR and RT-qPCR on key operational days (days 0, 15, 30, 60, 90, 120, and 180). Quant Studio S3 (Fisher Scientific, Waltham, MA, USA) equipment was employed to perform the experiments.

The reaction mixture of 25 µL, was composed of: 19.36 µL DEPC sterile water, 2.5 µL buffer with MgCl_2_, 0.5 µL dNTPs, 0.15 µL forwards and reverse primers at 10 µmolar, 0.125 µL Taq polymerase, 0.125 µL SYBR Green, and 0.0625 µL BSA (BioLabs). The primers used are shown in Table S1. For RT-qPCR, the reverse transcription of RNA to cDNA was performed before qPCR amplification, following the protocol defined by Maza-Marquez et al. [15]. Standard curves were calculated using ten-fold dilutions of linearised plasmid-carrying inserts of target genes.

### 2.8. Statistical Analysis

The diversity analyses were calculated using PAST v3.4. software. The α-diversity was evaluated by means of Simpson and Shannon–Wiener indices. The β-diversity, analysed to compare the differences between pairs of samples, was assessed by the Whittaker index.

The similarity percentage analysis (SIMPER) was calculated to observe the contribution of dominant OTUs to dissimilarity between pairs of samples of the control reactor and amended with the PC reactor. The OTU tables, calculated by a centred logarithm for the Bacteria domain, were used for calculation of SIMPER through the Bray–Curtis similarity using PAST software v3.4.

Principal coordinates analysis (PCoA) weighted by Bray–Curtis, was calculated through the centred logarithm OTU tables, and 999 bootstraps, in order to build the plot by PAST software v3.4.

### 2.9. Multivariate Redundancy Analysis and PERMANOVA

Multivariate redundancy analyses were conducted to detect the linkage between bacterial OTUs, biological samples over operational time, physico-chemical parameters (removal of COD, BOD, TN, settling velocity, granule size, MLSS), and the number of copies of cDNA and DNA target genes and the relationships between domains. The RDA was calculated with OTU tables corrected for zero values, centred log-ratio transformed and computed by 499 unconstrained Monte Carlo simulations under a full permutation model and run using the software CANOCO 4.5. The relative abundance of the bacterial phylotypes with at least ≥2.0% in biological samples on operational days 0, 15, 30, 60, 90, 120, and 180 were taken for the calculation procedure.

The PERMANOVA analyses were calculated to indicate the effect of pharmaceutical compounds on the most abundant bacteria (total and active), the copy number of target genes, and the physico-chemical parameters of the granular sludge systems. The analysis was computed using PASTv3 calculated under 9999 permutations of the Bray–Curtis algorithm [22].

## 3. Results and Discussion

### 3.1. Granular Development and Biomass Characteristics

**Figure 1:**
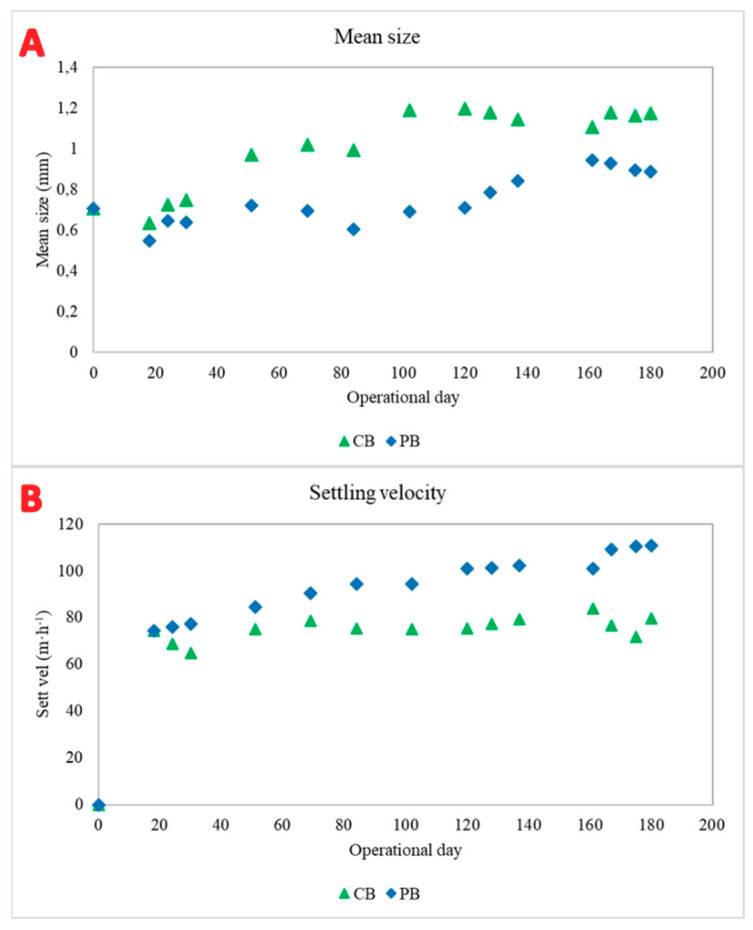
Mean size (**A**) and settling velocity (**B**) of granular biomass grown in CB (green triangle) and PB (blue diamond).

The bioreactors were inoculated with mature granules from a sequential batch reactor operated under a steady-state period at 15 ± 1.2 °C. The average size of granules used for the inoculum was around 0.70 mm. During the first twenty days of operation, both granular biomasses showed a reduction in diameter, with values of 0.57 mm and 0.60 mm for the CB and PB, respectively (Figure 1A). Then, the trend altered from operational day 50, especially in the CB, because the mean size of the granules enlarged until they reached average values ranging from 1.00 to 1.20 mm. The size of granular particles cultivated in the CB remained stable from operational day 110. The largest granules were detected in this reactor, even larger than the granules used for the inoculum. Thus, these results demonstrate the essential role of the design parameters of the bioreactor (for instance height–diameter ratio) and influent characterisation for the granule conformation, agreeing with Awang et al. [29].

In the PB, the granular size decreased during the first month of operation, from 0.70 mm to 0.55 mm, possibly caused by the effect of pharmaceuticals on the microbial population. Then, there was a weakly rising trend from operational day 50 to operational day 140. After this period, the granules were very stable and dense, with average values between 0.80 mm and 1.00 mm. Likewise, granules of the PB exhibited a compact structure with a spherical outer shape. Wan et al. [30] reported an increase in EPS production in the presence of pharmaceutical compounds, suggesting a stronger aggregation of cells.

In general, the dimensions of the granular biomass were higher in the CB, while those in the PB had a smaller size, albeit with a more compact and denser aspect (Figure 1A).

The granular properties were also measured by the settling ability of the biomass (Figure 1B). This parameter showed an opposite trend in comparison with granular size, because the smaller granules settled faster. Consequently, the biomass belonging to the PB settled faster in general terms, a fact previously described by Wan et al. [30]. These results suggest that the pressure exerted by the chemical compounds on the biomass makes the granules denser, so they can decant faster, improving the settling rate and their physical strength. On the other hand, the granules from the CB showed a larger size but a slower settling velocity, around 10 m h^−1^ slower than the granules in the PB.

The mean size and settling velocity analyses showed that there was a negative correlation between both parameters. The fastest granules were found in the PC and reached average velocities that exceeded 100 m h^−1^. Biomass from the CB had a settling velocity ranging from 65 to 82 m h^−1^.

**Figure 2:**
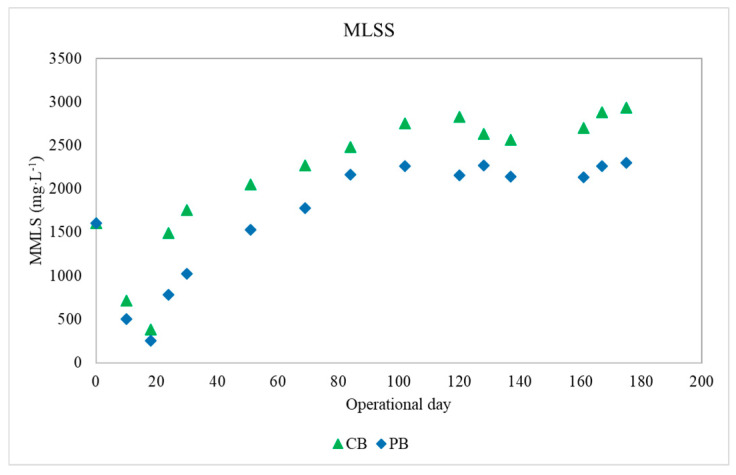
Biomass concentration expressed as mix liquor suspended solids (MLSSs) for CB (green triangle) and PB (blue diamond).

The seed granular sludge used as an inoculum in the reactors had an MLSS concentration of 1.6 g L^−1^. In the first few days, the MLSSs were reduced from 1.6 to 0.26 g L^−1^ due to the effluent withdrawal in the reactors (Figure 2). This pattern is widely observed during the setup of AGS systems and is caused by microbial selection by the operational conditions [31]. After 20 days of operation, the MLSSs increased sharply from 0.9 g L^−1^ to 2.2 g L^−1^ in the PB reactor, while that in the control bioreactor reached 3.1 g L^−1^. Therefore, MLSSs experienced a strong washout after 25 days of operation, as described by Othman et al. [32], presumably because of the transition from one bioreactor to the other, design bioreactor. The lower MLSS concentration in the PB could be caused by the presence of drugs in the influent water, according to findings by Wan et al. [30], in which the MLSS concentration was slightly lower in the reactor amended with antibiotics than in the control reactor.

### 3.2. Physico-Chemical Removal Efficiencies

**Figure 3:**
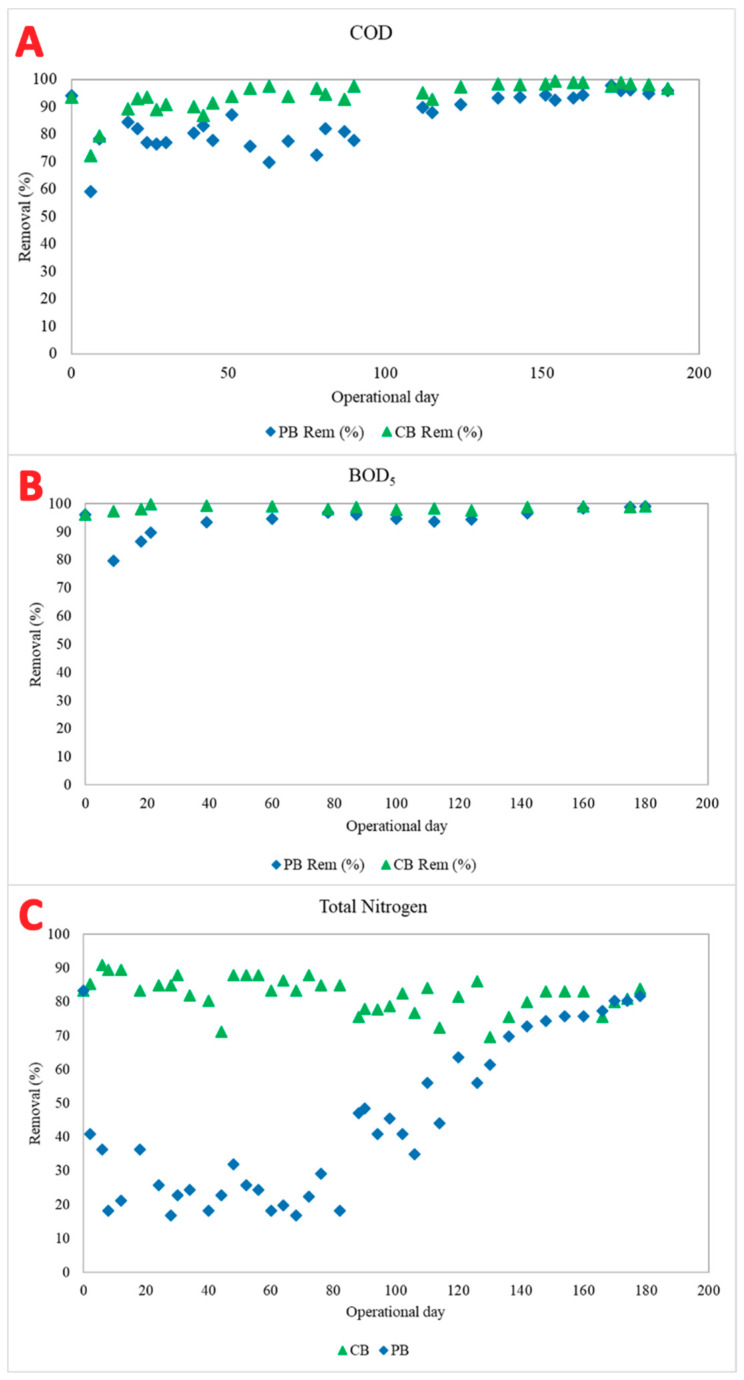
Chemical oxygen demand removal ratio (**A**); biological oxygen demand removal ratio (**B**); and nitrogen removal (**C**) for the control reactor (green triangle) and pharmaceutical reactor (blue diamond).

The physico-chemical performance ratio was evaluated in the CB and PB. At the beginning of experimentation, COD removal values showed a decreasing trend, while they seemed higher from operational day 20 onwards, reaching a steady-state scenario. This fact was linked with the strong depletion of MLSSs in the system due to the selection of floc-forming microorganisms. The differences between the pharmaceutical and control reactor were more strikingly marked until operational day 100, because in this period the CB reactor was able to remove 95% of COD, while the PB removal efficiency was in the range of 70 to 80%. From operational day 100, a stable scenario was achieved for both reactors regardless of the pharmaceutical compounds added, the highest efficiency being close to the range of 93 to 98% (Figure 3A).

The BOD_5_ removal followed a similar trend to the COD removal efficiency (Figure 3B). During the first month, BOD_5_ concentration in the effluent was around 100 mgO_2_ L^−1^ in the PB. Then, the removal ratio steeply increased, with values in the effluent of less than 15 mgO_2_ L^−1^ in both bioreactors. Despite both reactors achieving very good effluent quality, the PB generated an effluent with slightly higher BOD_5_ concentrations.

The nitrogen removal trend was significantly different amongst the reactors (Figure 3C). The control reactor remained stable from the beginning to the end of experimentation, with a performance displaying values always higher than 80% of total nitrogen, except for occasional days with a removal ratio between 60 and 70%. These results can be contrasted with the values obtained from the nitrogen analysis of the PB reactor, which showed a robust effect in the performance related to total nitrogen removed, due to low rates of nitrogen removal within the system. The average values of the removal ratio were lower than 50% until operational day 110. Some studies have reported the effects that pharmaceuticals had on ammonia oxidation as well as in denitrification processes [26]. Thus, it was reasonable to speculate that not only nitrifying bacteria, but also some other microbes involved in parallel metabolic processes, would be affected. Then, the tendency of nitrogen removal grew progressively until reaching stable values, higher than 70%, at operational day 140.

The results of total nitrogen removal showed a clear effect of pharmaceutical compounds on the nitrogen cycle carried out by the granular biomass, because these chemicals were the only difference between both reactors. In this sense, it is possible to conclude that the required time to achieve good removal ratios in a reactor treating pharmaceutical compounds is longer, but after a period of acclimatisation, the ratios are similar with respect to a non-pharmaceutical scenario.

In Figure S2, it is possible to observe in more detail the differences amongst reactors in the ammonium oxidation and denitrification processes. The CB was very stable; the ammonium oxidation ratio was excellent from operational day 80 to the end of the experiment with practically all the ammonium in the effluent oxidised to nitrite. Nitrite and nitrate conversion was effective, with nitrate values lower than 8.0 mg L^−1^ and nitrite concentrations lower than 4 mg L^−1^. On the contrary, the ammonium oxidation was not so good in the PB, although the trend was positive. Therefore, the denitrification process was not successful until operational day 120, from which values of 20 mg L^−1^ were reached.

### 3.3. Pharmaceutical Compound Analysis

**Figure 4:**
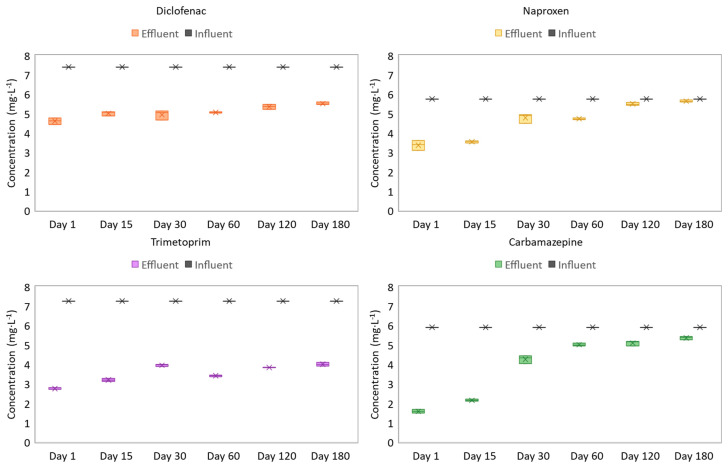
Effluent and influent concentrations of pharmaceutical compounds added in PB.

Four drugs were chosen as target pollutants, namely: diclofenac, naproxen, carbamazepine, and trimethoprim. The profile of pharmaceutical compound analysis is shown in Figure 4, reproducing the influent and effluent concentrations of the AGS system. For each contaminant, the influent concentration was 25 µM.

The results showed that the PB had a better removal effect on two drugs, trimethoprim and diclofenac, while the removed concentration of naproxen and carbamazepine was lower. In general terms, at the beginning of experimentation, the effluent concentration was very low, removing 30 to 70% of the influent concentration. This fact could be explained by the adsorption in the external layers of the AGS caused by the superficial charges of EPS [33,34]. However, over operational time, the concentration measured in the effluent showed lower values of biodegradation, biotransformation, and/or bioadsorption by the granular sludge. Therefore, a gradual decline of pharmaceutical removal was observed, probably related to the decrease in AGS adsorption capacity, especially for carbamazepine and naproxen. This hypothesis has been described previously by several authors researching AGS technology for the treatment of pharmaceutical compounds [34,35]. Moreover, other authors presented the AGS system as more able to degrade some pharmaceuticals such as sulfametazol, while other pharmaceuticals, such as roxithromycin, are removed from the effluent by adsorption mechanisms of AGS biomass [36].

As was mentioned before, carbamazepine was the most recalcitrant compound in the effluent despite the high capacity of AGS to biotransform, biodegrade, and/or bioadsorb pharmaceuticals [34,37]. This effect was previously described by Carballa et al. [38], who found carbamazepine to be highly resistant to degradation, while diclofenac showed moderate to high removal in wastewater treatment plants. Likewise, Blunt et al. [39] reported that trimethoprim has been proven to be highly resistant to degradation through wastewater treatment processes. Subsequently, this fact pointed out and made stronger the hypothesis about the bioadsorption ability of AGS to capture trimethoprim. Moreover, Barros et al. [40] demonstrated great variations of granular biomass to remove trimethoprim in wastewater, varying from 0 to 80%. The results of this experimentation showed that naproxen was poorly removed, especially at the end of the experiment, from operational day 120 to 180, possibly due to the saturation of the biomass by the different pharmaceuticals existing in raw water. Other authors have highlighted a better removal capacity for anti-inflammatory drugs, such as diclofenac or naproxen, than antibiotics such as trimethoprim, or antiepileptic drugs such as carbamazepine [41]. It is important to mention that the granular conformation was not greatly affected by the presence of pharmaceuticals, although some significant differences were found in comparison with the CB, mainly in the size and the settling ability. Thus, it was noted that the presence of pharmaceuticals made the granules more compact and denser than without the presence of drugs [30].

### 3.4. Abundance of Total and Metabolically Active Populations of Bacteria, Archaea, and Fungi in Aerobic Granular Sludge Reactor

**Figure 5:**
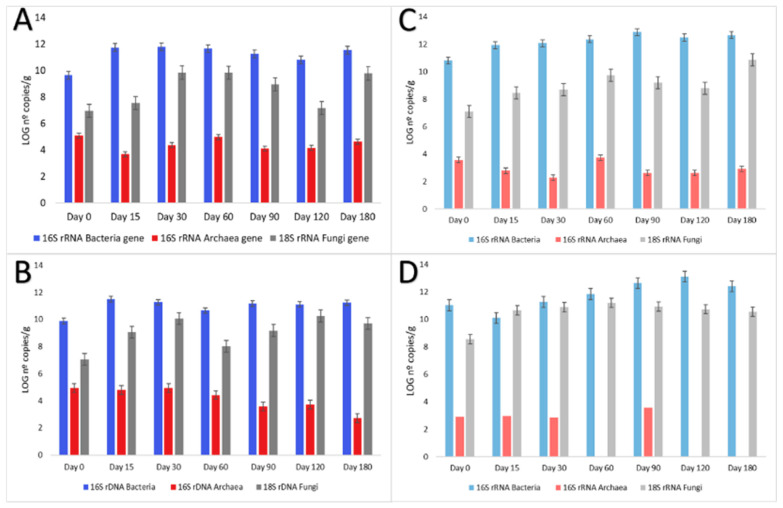
Number of copies of 16S rRNA gene of bacteria and archaea, and 18S rRNA gene of fungi per gram of granular biomass of the control bioreactor during the whole experimental period (**A**); number of copies of 16S rRNA gene of bacteria and archaea, and 18S rRNA gene fungi per gram of granular biomass of bioreactor amended with pharmaceutical compounds during the whole experimental period (**B**); number of copies of 16S rRNA of bacteria and archaea, and 18S rRNA fungi per gram of granular biomass of the control bioreactor during the whole experimental period (**C**); number of copies of 16S rRNA of bacteria and archaea, and 18S rRNA fungi per gram of granular biomass of bioreactor amended with pharmaceutical compounds during the whole experimental period (**D**).

To quantify the target genes in the granules, RT-qPCR and qPCR were conducted (Figure 5). The absolute abundance of total bacteria increased strongly between operational day 0 and day 15. Then, these genes remained tangibly stable from operational day 15 for both bioreactors, with an order of magnitude of around 12. For target genes of total archaea, the quantification showed slight and progressively decreasing values over the operational time, because at the beginning of the experiment, they were reported to be close to an order of magnitude of five, while those at operational day 180 had orders of magnitude of 4 and 2, for CB and PB, respectively. Therefore, total archaea genes were considerably affected by the presence of pharmaceutical compounds, possibly because they had competitive disadvantages against bacterial or fungal microorganisms. Hence, archaea suffered a decrease during the operation, a fact that could be corroborated with quantification of the activity in Figure 5D, since archaea activity disappeared from operational day 30. On the contrary, Figure 5C shows a loss of archaeal activity but not the complete disappearance of this domain, a fact observed without the presence of PCs. The results reflect the importance of evaluating the total and active microorganisms, because they provide an overview of the abundance and activity in which the target microorganisms are involved in pollution degradation, as Maza-Marquez et al. [16] reported.

The trend of fungi is very variable, as Muñoz-Palazón et al. [22] and Maza-Marquez et al. [15] have reported, because it depends closely on the environmental conditions and operational parameters. In this sense, the total and active quantification provided evidence about the fungal capacity to counteract toxic compounds [3]. In this way, there was a markedly larger number of copies of active fungal organisms than total fungi of the order of one or two magnitudes, regardless of the reactor. Therefore, the fungal copies seem to have the capacity to resist pharmaceuticals because the number of copies was larger in the PB (Figure 5B,D). In general, the number of copies of 18S rRNA and rRNA genes weakly increased over the experimental time until granules reached two orders of magnitude of fungi per gram of biomass.

### 3.5. Aerobic Granular Sludge Prokaryotic Composition of Total and Active Population Analysed by Massive Parallel Sequencing

#### 3.5.1. Dynamics of Total and Active Metabolically Bacterial Communities

**Figure 6:**
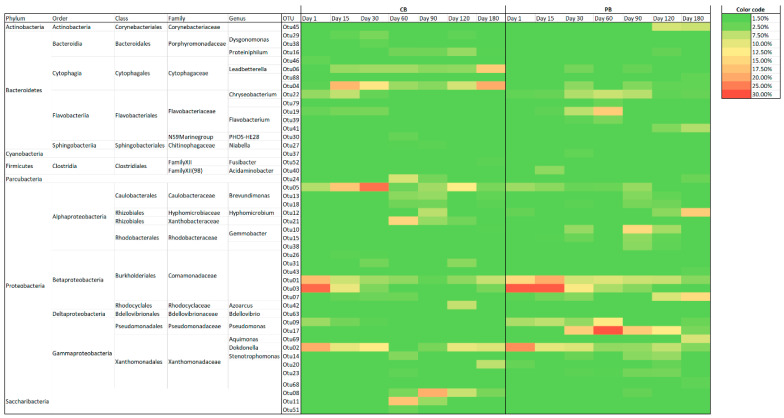
Distribution of the microbial community of DNA (>1.50%) in the reactors CB (control) and PB (supplemented with pharmaceuticals).

The bacterial community established within the granules plays a crucial role in the physico-chemical performance and granular conformation, which could be affected by the presence of emergent contaminants, such as pharmaceuticals, as reported by Amorin et al. [35]. Obviously, the bacterial communities of granules used as inocula were very similar for both reactors at operational day 1. The most dominant OTUs were taxonomically affiliated to the *Comamonadaceae* family and the *Dokdonella* genus. Several phylotypes belonging to the *Comamonadaceae* family have been commonly reported in aerobic granular sludge (Figure 6). The family is able to carry out metabolisms involved in the nitrogen cycle and organic matter degradation [17]. Nevertheless, *Dokdonella* is mostly described in aerobic granular sludge systems operated at low temperature [17,42]. The genus *Dokdonella* contains several known species and some of them have physiological characteristics that reduce nitrate in aerobic conditions [43]. Several technologies, such as activated sludge, have shown the *Dokdonella* genus and the *Comamonadaceae* family to be among microorganisms involved in the degradation of nitrate-containing organic pollutants [17,43]

The total bacterial community in the control reactor showed a decline in relative abundance of dominant OTUs from the inoculum. The decrease could be caused by changes in operational conditions, and especially in the reactor configuration and design, in comparison with the granules used as an inoculum processed by the reactor. Consequently, other phylotypes proliferated from operational day 15 to 30, with some OTUs belonging to the *Cytophagaceae* family and the *Brevundimonas* genus being especially interesting. The number of representative OTUs with high relative abundance changed over the operational period, although some of them, such as the *Cytophagaceae*, *Dokdonella*, *Brevundimonas* and *Comamonadaceae* families, remained stable. At days 60 and 90, together with those previously described, the most representative bacteria were taxonomically classified as belonging to the *Saccharibacteria* phylum. At the last stage of operation, the bacterial community study of the CB showed that the community remained in equilibrium in relation to previous days, demonstrating the stability of the bacterial community.

On the other hand, the reactor amended with pharmaceuticals reflected a considerable influence on the bacterial community in comparison with populations of the CB. Then, Otu001 was present during the whole experiment, although its relative abundance was lower over time. The major changes in the population were reported from operational day 30, possible due to the acclimatisation of microorganisms to the presence of toxic compounds. In this sense, the diversification of phylotypes was tangible because several phylotypes acquired relevance in the population, such as Otu009, Otu010, Otu017, and out019, OTUs taxonomically affiliated to *Pseudomonas*, *Gemmobacter*, and *Flavobacterium.* Certainly, the majority of them have been reported in biological technologies for treating wastewater containing pharmaceuticals [35,40]. The *Flavobacterium* genus could play an essential role in the treatment of drugs because it possesses antibiotic resistance genes [39]. In fact, Tiwari et al. [44] reported *Flavobacterium* growth in the presence of pharmaceuticals. Moreover, *Flavobacterium* was reported to promote the production of cyclic-diguanylate, which encourages EPS production and, in that way, protects microorganisms within granules against toxic compounds [35], while this production stimulates the compaction and aggregation of cells [11]. In the steady state, other OTUs belonging to the genera previously described proliferated. At the end, the most dominant bacteria were Otu007, belonging to the *Comamonadaceae* family, as well as *Hyphomicrobium* which had significant representation in the population, with more than 16.00% of relative abundance. *Hyphomicrobium* has been reported as the most abundant phylotype in an aerobic granular sludge system treating pharmaceutical compounds [36].

**Figure 7:**
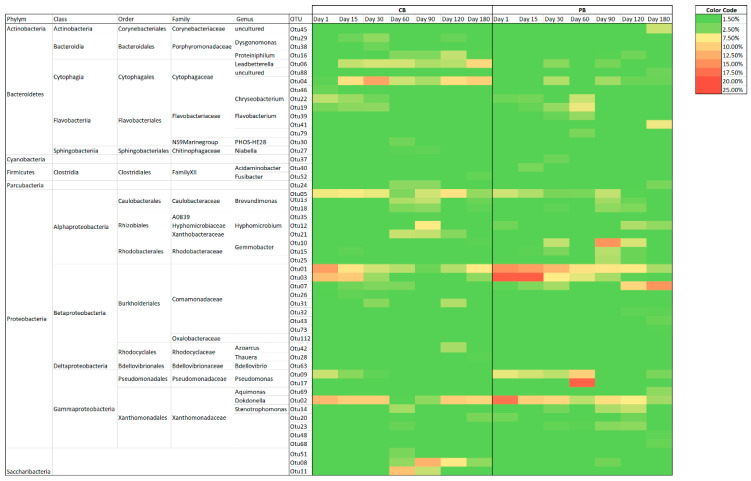
Distribution of the microbial community of RNA (>1.50%) in the reactors CB (control) and PB (supplemented with pharmaceuticals).

The active bacterial population followed a similar dynamic for both the CB and PB (Figure 7). Undoubtedly, the trend was sharper, meaning that the relative abundance was higher for the most representative phylotypes. The CB showed high relative abundance of some newly described OTUs, especially in the case of *Leadbetterella* and *Xanthobacteraceae,* as well as other phylotypes belonging to *Saccharibacteria* and *Brevundimonas*. A similar trend was observed for the PB, because the representative OTUs acquired a higher relative abundance in the dynamic populations.

#### 3.5.2. Archaeal Population

The archaeal population was studied over operational time, however, the results obtained by the taxonomic affiliation with two different databases (full SILVA and MiDAS) did not provide information about the taxonomy of them. The lack of knowledge about the archaeal community should promote the development of deeper studies about this domain and its ecological role in biological wastewater, which has been reported to have essential roles in the natural environment and in engineered systems [17].

Some archaea found at a low relative abundance were *Methanoperedens* and *Methanobrevibacter,* as well as some OTUs belonging to *Euryarchaeota*.

### 3.6. Alpha and Beta Diversity Analyses

The values of the Simpson and Shannon–Wiener indices for bacterial populations from the inoculum to the end of experimentation, with the aim to evaluate the diversity and evenness of the biological samples, are shown in Table S2. The results of DNA analyses reflected analogous values for both bioreactors over the operational time. On the contrary, the Shannon–Wiener results demonstrated a rise in diversity and evenness in the CB from operational day 0 to day 15, which remained stable until the end of operation, while weak oscillations were observed in the PB. The analysis could be completed with community studies because a lower evenness was observed in the PB. In this way, during the first 3 months of operation, the PB showed lower evenness and diversity indices in the bacterial communities. Nevertheless, after this period, they increased notably, achieving similar values as in the control bioreactor. This trend could be explained by the acclimatisation of bacterial communities to the presence of toxic compounds in the influent, as well as the biodegradation, biotransformation, and/or bioadsorption of pharmaceuticals.

The β-diversity analysis is shown in Figure S3 for total bacteria (A) and metabolically active bacterial populations (B). The Whittaker index for DNA and RNA showed a high similitude of the CB at day 180, with all samples belonging to this reactor despite the operational time. However, greater dissimilarities were found in the progressive biological samples of the PB over the experimental time, showing changes produced by the operational conditions and factors, such as the pharmaceutical compounds. In fact, the most pronounced differences were observed in the pairs of samples belonging to days 60, 90, and 120, when the system started to achieve physico-chemical stability.

Likewise, a striking pattern between reactors was detected, with samples at day 180 of the CB showing high similarities with all samples of the PB reactor. This fact could mean that generic phylotypes that comprised the mature granules are extremely stable.

### 3.7. Similarity Analysis of Bacterial Samples

The SIMPER analysis showed the contribution to dissimilarities between dominant and representative phylotypes for both reactors, indicating the relative impact of each OTU (Figure S4). The number of total OTUs that contributed more than 1.50% to RNA or DNA samples totaled 19. The general trend showed that the contributions greater in samples of metabolically active genes than in the total 16S rRNA genes. One representation of the trend highlighted Otu003, which showed the highest contribution to disparities between reactors, with 6.00% and 8.00% of relative abundance for DNA and RNA samples, respectively. These outcomes suggested that despite the existence of specialised phylotypes, metabolically active microorganisms modified the relationships in the communities. Therefore, this hypothesis was reinforced by several OTUs, which were found to have a great contribution to dissimilarities in samples of 16S rDNA, but they were even more marked in the metabolically active populations. Otu003 belonged to the *Comamonadaceae* family and was one of the most representative phylotypes in both systems, originating from the biomass used as an inoculum, whereas, during the operation, Otu003 was more persistent in the PB.

Additional OTUs that contributed to disparities with more than 5.00% of relative abundance were Otu001, Otu002, and Otu004, taxonomically affiliated to the *Comamonadaceae* family, the *Dokdonella* genus, and the *Cytophagaceae* family, respectively. These OTUs showed opposite trends for both reactors, as corroborated by the community studies. The results showed *Cytophagaceae* as exclusively outstanding in the CB, and their metabolic activity consolidated pronounced differences (>7.00%) related to the PB, which did not show the presence of Otu004.

Otu006, Otu007, Otu008, Otu009, Otu010, and Otu17 contributed to dissimilarities between the CB and PB. Of special interest were Otu010 and Otu17, belonging to the *Gemmobacter* and *Pseudomonas* genera, because they contributed to differences at 4.00%. These phylotypes were dominant in the PB over the operational time under drug loads.

On one hand, principal coordinate analysis (PCoA) for 16S rRNA gene samples revealed a strong clustering of all samples of the CB, and the samples of the first 3 months of operation of the PB, while samples of mature granules of the PB reflected more distance between themselves (Figure S5A). On the other hand, the PCoA plot shown in Figure S5B reflects a larger spatial distribution of bacterial 16S rRNA samples than 16S rRNA gene samples. One big group was clustered with the initial samples of both AGS reactors; separately, a second aggrupation was found at day 90 and day 60 of the CB, where some phylotypes such as *Gemmobacter* and *Pseudomonas* notably proliferated. Finally, a third group was composed of samples at day 60 and day 180 of the PB. The results demonstrated that drugs affected the total population and, more mildly, the metabolically active bacterial population.

### 3.8. Multivariate Redundancy and PERMANOVA Analyses

**Figure 8:**
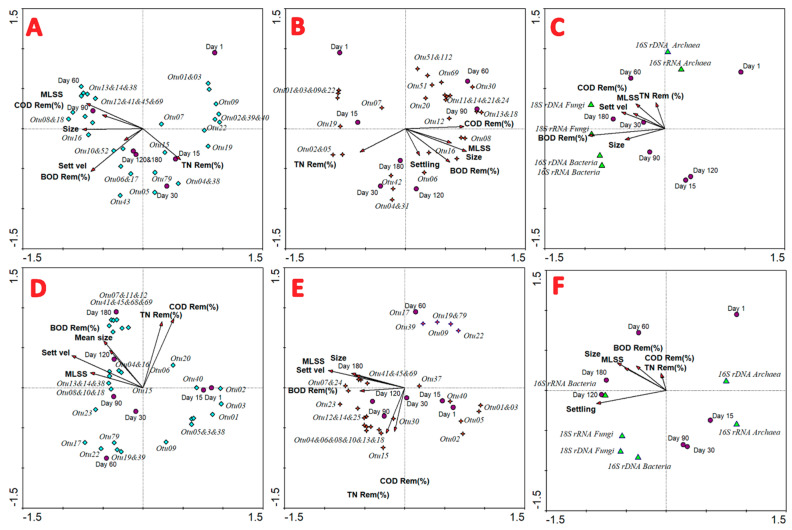
Multivariate redundancy analysis of the most abundant 16S rRNA bacterial genes for CB (**A**) and PB (**D**) with physico-chemical removal ratio (logarithm); multivariate redundancy analysis of the most abundant 16S rRNA bacterial genes for CB (**B**) and PB (**E**) with physico-chemical removal ratio (logarithm); multivariate redundancy analysis of total and metabolically active copies of target genes with physico-chemical performance for CB (**C**) and PB (**F**).

The RDA shown in Figure 8A reflects the correlations of the most dominant OTUs with >2.00% of relative abundance with the physico-chemical removal performance and granular conformation in the CB reactor. The total nitrogen removal was linked with Otu004 and Otu038 which are taxonomically affiliated to the *Cytophagaceae* and *Rhodobacteriaceae* families. *Rhodobacteraceae* is an enormous and complex family whose members have been reported as aquatic bacteria frequently thriving in marine environments and wastewater [45]. Enrichment of this family in biological reactors for nitrogen removal has been previously reported [46], as well as some genera of this family, such as *Zooglea*, playing a crucial role in the conformation of granular sludge [17]. Likewise, the *Cytophagaceae* family is closely associated with nitrogen fixation metabolism as nitrogen-incorporating bacteria in several environments [47]. The COD removal ratio, MLSSs, and the granular size were positively correlated with several OTUs, and among them the most representatives in the CB were *Hypomicrobium*, *Brevundimonas*, *Flavobacterium*, *Xanthomonadaceae*, and *Dysgonomonas*. The majority of them can degrade organic matter and also carry out other complementary metabolisms, such as heterotrophic denitrification in the case of *Hypomicrobium* [48]. Furthermore, they produce extracellular polymeric substances, assisting cell aggregation [11]. The settling velocity and BOD_5_ degradation were strongly correlated with the *Leadbetterella* genus that has been suggested as a group of functional syntrophic organic compound-degrading bacteria [49].

The RDA displayed in Figure 8B indicates the pronounced disparities found in the active population and their relationships with the performance in the CB. In this sense, the total nitrogen removal ratio was strongly linked with Otu002 and Otu005, affiliated to *Dokdonella* and *Brevundimonas*, respectively, which are reported as nitrogen removal bacteria in wastewater environments [50]. For instance, both genera reduce nitrate in aerobic conditions [51,52]. On the other hand, the organic matter removal (COD and BOD_5_ removal ratio), as well as biomass properties (MLSSs, size and settling ability), were positively correlated between them. This cluster was interconnected with *Saccharibacteria*, *Proteiniphilum*, *Leadbetterella*, and *Arzoarcus* genera. Certainly, some of these taxa are unusual in wastewater, but they have been reported previously [53].

In order to compare the total and active population linked with removal performance and granular properties in a bioreactor amended with PCs, multivariate redundancy analysis was calculated, which is shown in Figure 8D,E. Figure 8D shows the total bacterial population and the correlation with the physico-chemical determinations. The analysis reveals the linkage of biological samples at days 90, 120, and 180, positively correlated with higher removal ratios, as well as larger granular size and biomass concentration. The number of OTUs positively correlated to this parameter is small, so the majority of them are negatively correlated with high performance, such as Otu001, Otu002, Otu003, Otu005, and Otu009. These results show that the initial population from the inoculum is not able to acclimatise to the presence of a large load of pharmaceuticals, and thus encourages the proliferation of bacteria able to resist the presence of these compounds. Consequently, the OTUs positively correlated with high removal ratios and granular stability were Otu007, Otu011, Otu012, Otu016, Otu041, Otu045, Out068, and Otu069, among others. A lot of them proliferated during the operation under pharmaceuticals, such as *Corynebacteriaceae* or *Comamonadaceae*, which attained a very high relative abundance at operational days 120 and 180. These genera are common in aerobic granular sludge systems [12,17,21].

In the same way, the RDA shown in Figure 8E follows a similar pattern, with analogous correlations with the RDA of total bacterial genes. In this sense, these results meant that in the presence of toxics, the total and metabolically active population are more closely linked, and thus they are more representative, and they are more deeply corroborated. Validating this fact, Otu041, Otu045, and Otu069 were tightly related to granular size, settling ability, and MLSSs. In the same direction, Otu001, Otu002, and Otu003 were strong and negatively correlated with the right operation and performance of the system.

The RDA associated the copy number of total and active target genes with the pollution degradation in the CB (Figure 8C) and PB (Figure 8F). For the CB, a correlation was found for the copy number of total and metabolically active archaeal 16S RNA genes and organic matter, nitrogen removal, and the granular properties. On the contrary, a strong and positive correlation was found for 18S rRNA gene fungal copies with COD and TN removal, as well as with MLSS concentration and the decanting velocity, especially close to the biological sample at day 180.

The reactor amended with pharmaceutical compounds showed a different pattern (Figure 8F), because, in this case, a negative correlation was detected for the high physico-chemical yield and granular stability with the copy number of total and active archaea. Thus, the outcomes of both RDA analyses were either negative or neutral in the presence of archaeal microorganisms, with a good performance in this kind of technology, regardless of influent characterisation. This fact could be caused by the competitive disadvantage of this domain against the high abundance of bacterial or fungal organisms.

In the PB reactor, the copies of active bacterial 16S rRNA were closely linked with the granular properties (MLSSs, settling velocity, and granular size), while the total number of bacteria was not strictly positively correlated. Likewise, the number of total and metabolically active fungal copies was positively correlated with the settling ability of granules. Some authors have described the role that fungi play in the initial microbial aggregation and optimum granular conformation, because they act as union bridges between cells which contribute to compactness [17,21].

PERMANOVA analysis allowed us to corroborate the statistically significant effect of the treatment of large loads of pharmaceutical compounds using aerobic granular sludge (Table S3). In this way, the PERMANOVA analysis calculated the influence of the drug mixture on metabolically active and total bacteria (the most representative ones). The values obtained marked the significant effect that pharmaceuticals have on Otu004 and Otu006 (ρ < 0.005), for both active and total bacteria. Additionally, in terms of activity, Otu008, which was affiliated with *Saccharibacteria* and only detected in the CB, was affected by pharmaceuticals.

On the other hand, all the physico-chemical determinations, except MLSSs, were significantly affected when treating pharmaceutical compounds. These results were corroborated in the chemical analysis and granular properties, as reflected in Section 3.1. and Section 3.2. This pattern was more strongly perceived during the first 100 days.

The absolute quantification of target genes was not affected by the pharmaceutical compounds, except for the active fungi, which increased significantly in terms of the number of copies in the PB. This fact could be supported by the competitive advantage that fungi have over bacteria in the presence of antibiotics.

## 4. Conclusions

Aerobic granular sludge systems were operated for the treatment of wastewater with a large load of mixed pharmaceutical compounds. The drug removal efficiency was especially marked for the anti-inflammatory diclofenac and the antibiotic trimethoprim. During the initial period of treatment, the granules showed a strong bioadsorption capacity. The granular conformation was not affected by the presence of pharmaceutical compounds due to high resistance capacity of the granules, although they were statistically significant in comparison with the control reactor in terms of settling velocity and size. The settling ability was improved by the presence of pharmaceutical compounds. High organic matter and nitrogen removal levels were reached after a period of acclimatisation, when the most resistant microorganisms, such as the genus *Hyphomicrobium* and *Comamonadaceae* family, played an important role in the system. The differences among bacterial communities were corroborated by SIMPER, PERMANOVA, PCoA, and β-diversity. The metabolically active communities in the bioreactor control and the bioreactor supplemented with pharmaceuticals evolved divergently. In the control reactor, the relative abundance showed similarity with the results of the total bacterial population, while the reactor amended with drugs showed a notable increase in relative abundance with respect to the development of the total community. Despite the high level of representation of some phylotypes in the DNA studies, their presence is not tightly correlated with the role microorganisms play. The qPCR and RT-qPCR showed the notable contribution of fungal organisms in the granular sludge technology with pharmaceutical compounds. Additionally, the absolute quantification confirmed the disadvantageous position of archaea in environments with high toxic loadings.

Thus, our research supplies essential information for the implementation of AGS systems at full scale to treat wastewater with high pharmaceutical compound loadings for urban or industrial wastewater. In this sense, we have provided a detailed study to demonstrate the effect of toxic compounds contained in wastewater on aerobic granular sludge systems based on chemical performance and the physical granular morphology, as well as the total and metabolically active microbial community within the granules.

## Data Availability

10.17632/65ghfmsbmw.1.

## Supplementary Materials

The following are available online at https://www.mdpi.com/article/10.3390/toxics9050093/s1, Figure S1: Configuration and design of AGS systems, Figure S2: Ammonium, nitrate and nitrite concentration in the effluent in control bioreactor (A) and pharmaceutical bioreactor (B), Figure S3: β-diversity analyses by Whittaker indices (A: DNA pair of samples; B: RNA pair of samples), Figure S4: SIMPER analysis of the most contribute OTU to dissimilarity between control reactor and reactor amended with pharmaceutical compounds, Figure S5: Principal coordinate analysis of total community (A); and metabolically active community (B) for control bioreactor (red) and amended bioreactor (purple), Table S1: (A) Primers used for quantification of total Bacteria, total Archaea, total Fungi throught RT-qPCR and qPCR. (B) qPCR conditions for the amplification and quantification of interest genes, Table S2: α-diversity analysis calculated thought, Shannon Wienner and Simpson indices for RNA and DNA biological samples for both reactors, Table S3: One way PERMANOVA analysis among control and pharmaceutical bioreactors.
