# Supplementary material for: Total and Metabolically Active Microbial Community of Aerobic Granular Sludge Systems Operated in Sequential Batch Reactors: Effect of Pharmaceutical Compounds
Source: Toxics. 2021 Apr 23;9(5):93. doi: 10.3390/toxics9050093 (PMC8146427; doi:10.3390/toxics9050093)
Supplement: Supplementary file 1 [file toxics-09-00093-s001.zip › toxics-1171976-supplementary.pdf]

Supplementary Materials: Total and Metabolically Active Microbial Community of Aerobic Granular Sludge Systems Operated in Sequential Batch Reactors: Effect of Pharmaceutical Compounds

Barbara Muñoz-Palazon, Aurora Rosa-Masegosa, Miguel Hurtado-Martinez, Alejandro Rodriguez-Sanchez, Ramiro Vilchez-Vargas, Alejandro Gonzalez-Martinez, Jesus Gonzalez Lopez

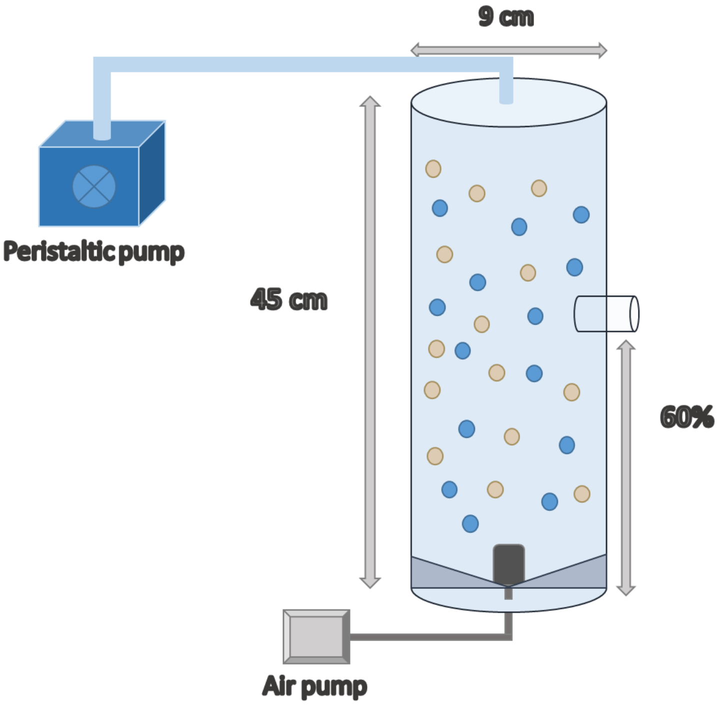

Figure S1. Configuration and design of AGS systems.

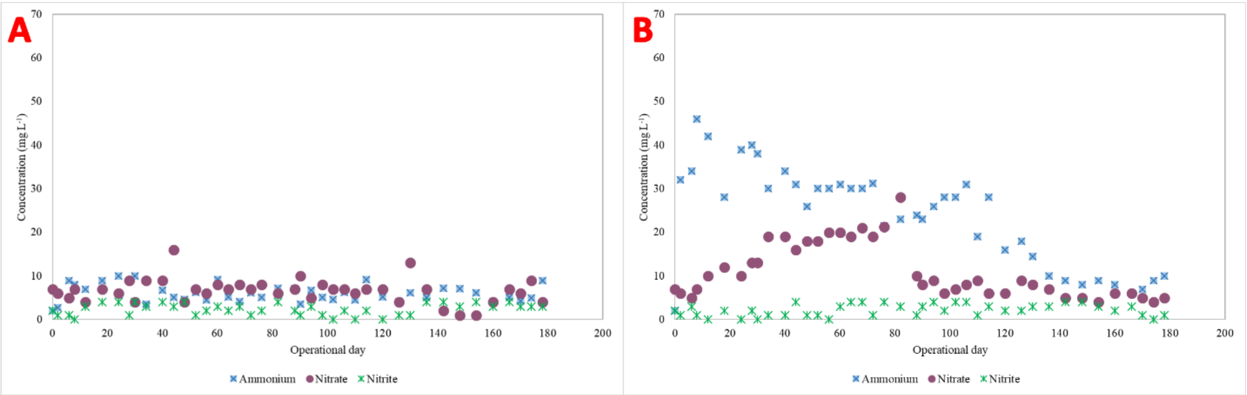

Figure S2. Ammonium, nitrate and nitrite concentration in the effluent in control bioreactor (**A**) and pharmaceutical bioreactor (**B**).

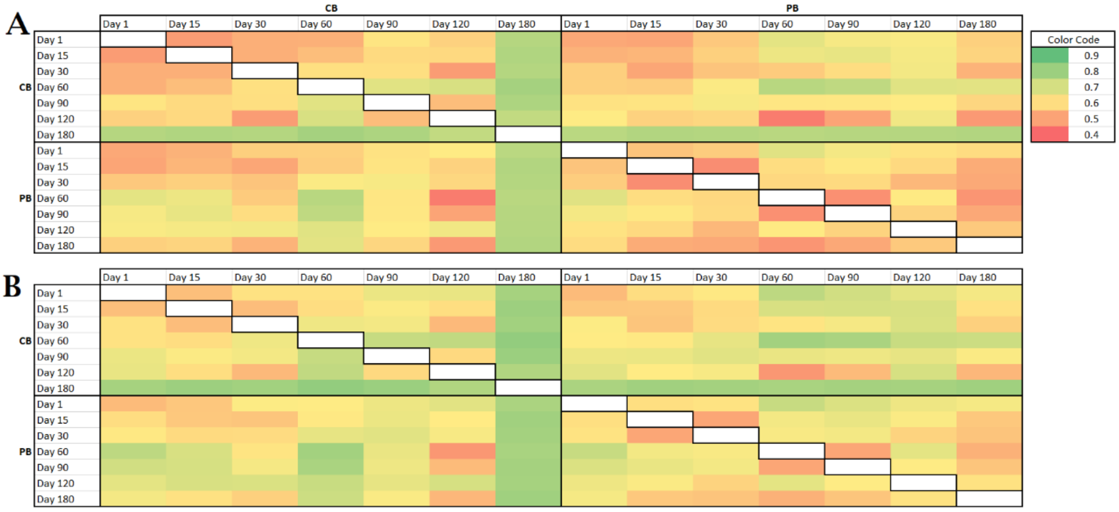

Figure S3. β-diversity analyses by Whittaker indices (A: DNA pair of samples; B: RNA pair of samples).

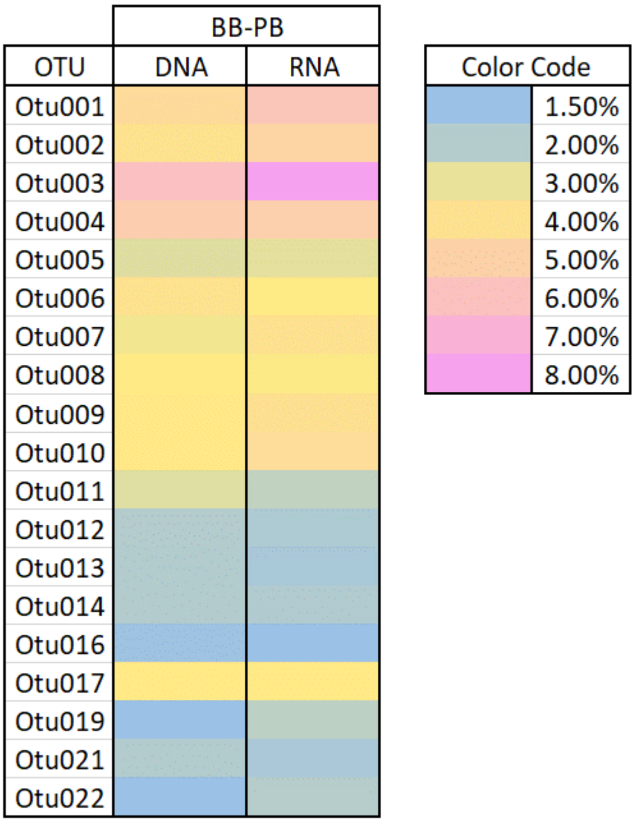

Figure S4. SIMPER analysis of the most contribute OTU to dissimilarity between control reactor and reactor amended with pharmaceutical compounds.

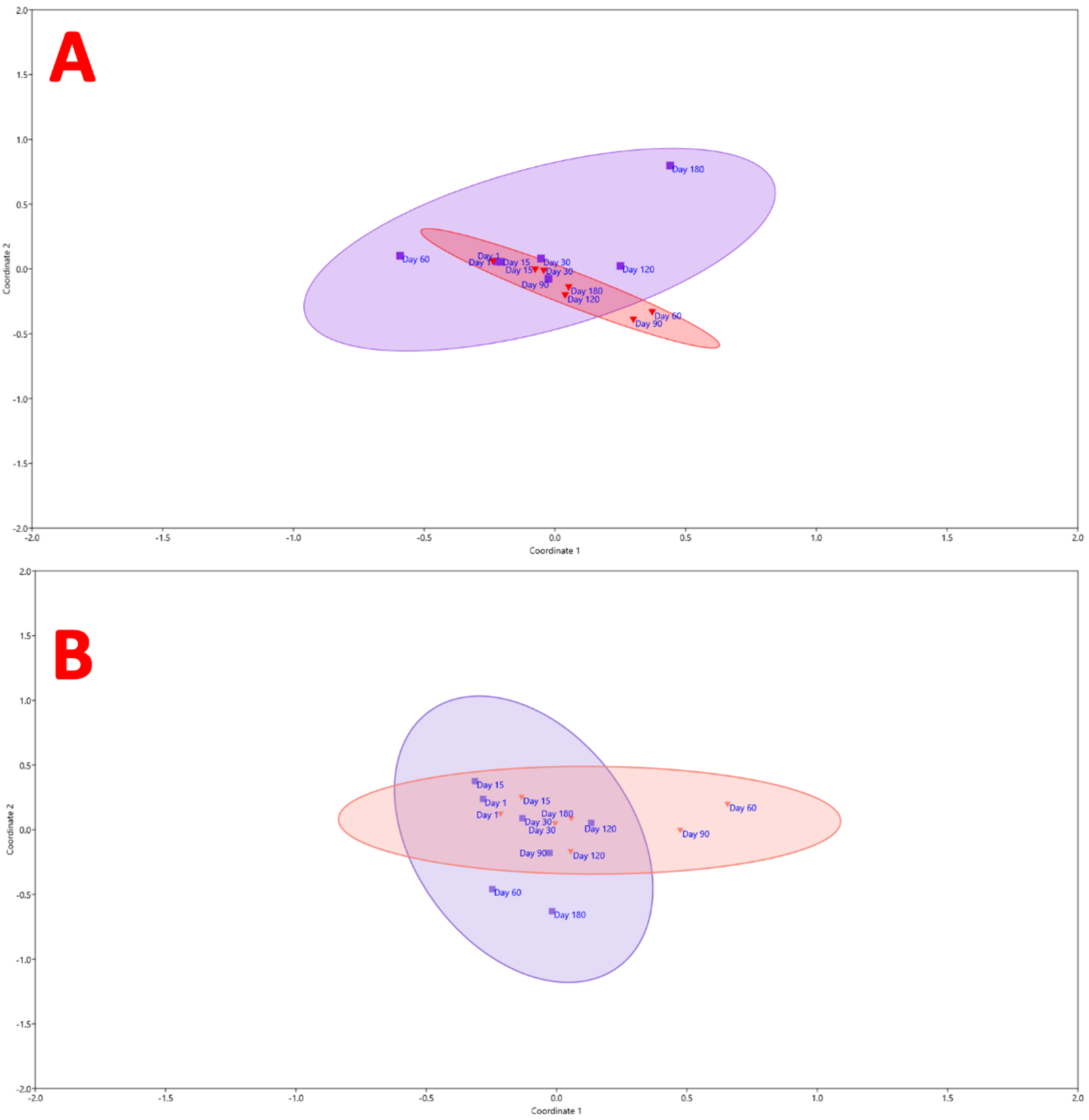

Figure S5. Principal coordinate analysis of total community (**A**); and metabolically active community (**B**) for control bioreactor (red) and amended bioreactor (purple).

Table S1. (**A**) Primers used for quantification of total Bacteria, total Archaea, total Fungi throught RT-qPCR and qPCR. (**B)** qPCR conditions for the amplification and quantification of interest genes.

A

Group	Gene Marker	Primers	Sequence (5′-3′)	References
Total Bacteria	V3-16S rRNA	P1 (341F)	CCTACGGGAGGCAGCAG	Muyzer et al., 1993
		P2 (534R)	ATTACCGCGGCTGCTGG	
Total Archaea	16S rRNA	ARCH915 (F)	AGGAATTGGCGGGGGAG-CAC	Yu et al., 2008
		UNI-b-rev (R)	GAC-GGGCGGTGTGTRCAA	
Fungi	18S rRNA	Fungi-QuantF	GGRAAACTCACCAG-GAGGTCCAG	Liu et al., 2012
		Fungi-QuantR	GSWCTATCCCCAKCACGA	

B

		Total Bacteria	Total Archaea	Total Fungi
	Initial denaturalization	95 °C, 7 min	95 °C, 7 min	95 °C, 3 min
Amplification (×40 cycles)	Denaturalization	95 °C, 30 s	95 °C, 30 s	94 °C, 30 s
	Primers annealing	60 °C, 30 s	60 °C, 30 s	62 °C, 30 s
	Elongation	72 °C, 30 s	72 °C, 30 s	62 °C, 45 s
	Melting curve	60 °C-95 °C + 2 °C/min. Fluorescence measured each 15 s		

Table S2. α-diversity analysis calculated thought, Shannon Wienner and Simpson indices for RNA and DNA biological samples for both reactors.

		DNA		RNA	
**Reactor**		**Simpson**	**Shannon**	**Simpson**	**Shannon**
CB	Day 1	0.921	3.712	0.910	2.902
	Day 15	0.952	4.132	0.927	3.001
	Day 30	0.951	4.131	0.929	3.087
	Day 60	0.967	4.488	0.945	3.229
	Day 90	0.960	4.210	0.940	3.179
	Day 120	0.955	4.082	0.928	3.004
	Day 180	0.959	4.420	0.926	3.065
PB	Day 1	0.916	3.674	0.869	2.574
	Day 15	0.920	3.784	0.894	2.853
	Day 30	0.958	4.195	0.937	3.173
	Day 60	0.905	3.775	0.893	2.687
	Day 90	0.946	3.924	0.927	3.002
	Day 120	0.972	4.578	0.946	3.286
	Day 180	0.966	4.208	0.932	3.192

Table S3. One way PERMANOVA analysis among control and pharmaceutical bioreactors.

rRNA	Otu01	Otu02	Otu03	Otu04	Otu05	Otu06	Otu07	Otu08	Otu09	Otu10
**F**	2.223	0.2388	0.6864	6.962	4.208	10.11	1.028	3.065	1.3	2.791
**ϱ**	0.140	0.776	0.464	0.009	0.0502	0.006	0.379	0.046	0.228	0.087
**rDNA**	**Otu01**	**Otu02**	**Otu03**	**Otu04**	**Otu05**	**Otu06**	**Otu07**	**Otu08**	**Otu09**	**Otu10**
**F**	1.714	0.3033	0.6197	3.866	2.185	3.619	0.964	1.322	1.761	2.791
**ϱ**	0.198	0.733	0.494	0.020	0.084	0.014	0.419	0.266	0.142	0.085
**Perfor-mance**	**BOD Rem (%)**	**COD Rem (%)**	**Mean Size**	**Settling velo**	**MLSS**	**TN Rem (%)**				
**F**	8.269	7.74	5.892	6.845	0.347	7.191				
**ϱ**	0.008	0.014	0.036	0.024	0.583	0.006				
**qPCR**	**16S rDNA Bacteria**	**16S rDNA Archaea**	**18S rDNA Fungi**	**16S rRNA Bacteria**	**16S rRNA Ar-chaea**	**18S rRNA Fungi**				
**F**	1.199	0.525	0.359	0.731	2.163	4.25				
**ϱ**	0.354	0.653	0.828	0.551	0.177	0.025				
